# Assessment of tumor motion reproducibility with audio‐visual coaching through successive 4D CT sessions

**DOI:** 10.1120/jacmp.v15i1.4332

**Published:** 2014-01-04

**Authors:** Samuel Goossens, Frédéric Senny, John Aldo Lee, Guillaume Janssens, Xavier Geets

**Affiliations:** ^1^ Center of Molecular Imaging, Radiotherapy, and Oncology (MIRO) Université Catholique de Louvain, Saint‐Luc University Hospital B‐1200 Brussels Belgium; ^2^ Montefiore Institute Université de Liège Liège Belgium; ^3^ ICTEAM Institute Université Catholique de Louvain Louvain‐la‐Neuve Belgium

**Keywords:** lung tumor motion, 4D CT, audio and visual coaching

## Abstract

This study aimed to compare combined audio‐visual coaching with audio coaching alone and assess their respective impact on the reproducibility of external breathing motion and, one step further, on the internal lung tumor motion itself, through successive sessions. Thirteen patients with NSCLC were enrolled in this study. The tumor motion was assessed by three to four successive 4D CT sessions, while the breathing signal was measured from magnetic sensors positioned on the epigastric region. For all sessions, the breathing was regularized with either audio coaching alone (AC, n=5) or combined with a real‐time visual feedback (A/VC, n=8) when tolerated by the patients. Peak‐to‐peak amplitude, period and signal shape of both breathing and tumor motions were first measured. Then, the correlation between the respiratory signal and internal tumor motion over time was evaluated, as well as the residual tumor motion for a gated strategy. Although breathing and tumor motions were comparable between AC and AV/C groups, A/VC approach achieved better reproducibility through sessions than AC alone (mean tumor motion of 7.2 mm±1 vs. 8.6 mm±1.8 mm, and mean breathing motion of 14.9 mm±1.2 mm vs. 13.3 mm±3.7 mm, respectively). High internal/external correlation reproducibility was achieved in the superior‐inferior tumor motion direction for all patients. For the anterior‐posterior tumor motion direction, better correlation reproducibility has been observed when visual feedback has been used. For a displacement‐based gating approach, A/VC might also be recommended, since it led to smaller residual tumor motion within clinically relevant duty cycles. This study suggests that combining real‐time visual feedback with audio coaching might improve the reproducibility of key characteristics of the breathing pattern, and might thus be considered in the implementation of lung tumor radiotherapy.

PACS number: 87

## I. INTRODUCTION

Tumor motion (TM) generates geometric uncertainty in the radiotherapy (RT) of lung tumors. It justifies specific strategies in the planning and delivery steps to ensure adequate target coverage through successive RT fractions. These strategies fall into two categories. The first one includes margin‐based approaches that either aim at covering the entire tumor trajectory derived from 4D information, such as the internal target volume technique (ITV),^(1)^ or take advantage of the geometrical time‐weighted mean tumor position derived the from midventilation (MidV)^(2)^ CT or the midposition (MidP)^(3)^ CT, extended with appropriate margins. The second category encompasses respiratory synchronized techniques that intend to minimize TM contributions in PTV margin calculation by monitoring the internal tumor position during respiration (breath‐hold, gating,^(4)^ tracking^(5)^). Tumor monitoring in these latter techniques can use external surrogates of the internal motion, to avoid the use of invasive procedures (implantation of radiopaque marker) and their potential complications.^(6)^ However, this approach requires a significant and stable correlation between the internal TM and its external surrogate, which is usually not the case when transient changes occur in the patient's breathing pattern.^(7)^

Whatever the RT strategy, improving reproducibility of the tumor position and motion through successive sessions is therefore desirable, particularly when treatment protocols do not involve online imaging for motion monitoring and treatment adaptation. In that regard, audio‐visual guidance might prove useful. Previous studies have already demonstrated that audio guidance stabilized breathing frequency^(8)^ and improved the external/internal correlation between the respiratory signal and the tumor position.^(9)^ Combining visual feedback with audio guidance further regularized both external breathing amplitude and baseline variation,^10^, ^11^ which strongly advocates the use of gated RT.^12^, ^13^ However, the impact of combining visual feedback with audio coaching on the internal TM itself, and on its reproducibility through successive sessions, has not been evaluated so far.

In this context, the present work aimed to assess the influence of an in‐house system of respiratory coaching on the internal TM reproducibility. The coaching included audio guidance, optionally complemented with visual feedback when the patients tolerated it. In particular, we studied the impact of the system on the reproducibility of some characteristics of breathing and TM, like the peak‐to‐peak amplitude, period, and signal shape, all measured through three to four separated sessions. The correlation between the external respiratory signal and internal TM over time was then evaluated, as well as the residual tumor motion (RTM) for a gated strategy. This study was performed on a series of 13 NSCLC patients treated by radical radiotherapy. They underwent repeated 4D CT acquisitions to evaluate the TM, while the respiratory signal was measured with magnetic sensors^(14)^ positioned on the epigastric region.

## II. MATERIALS AND METHODS

### A. Patient selection

Thirteen patients with primary stage I‐III non‐small cell carcinomas (NSCLC) were prospectively included in this study between June 2010 and September 2011. They were all referred to the radiotherapy department for either concomitant chemoradiation (CRT) or stereotactic body radiation therapy (SBRT). The internal review board approved this study and informed consent was obtained from all patients.

### B. Data acquisition

For all enrolled patients, the first session of 4D CT was part of their clinical planning protocol for RT treatment preparation. If the TM magnitude (in 3D) exceeded 5 mm, two or three 4D CTs were additionally acquired at the latest during the first week of the RT treatment, in order to avoid any radiation‐induced tumor modification. For all acquisitions, patients were immobilized in a thermoformed plastic mask (CIVCO Medical Solutions, Kalona, IA) in supine position, head first and arms along the body.

An audio‐visual coaching (A/VC) procedure was used in order to regularize the patient breathing through all imaging sessions. Before the first image acquisition, a training session was planned to record and characterize the respiratory pattern of each individual patient. The average frequency, the relative duration of inhalation and exhalation, as well as the amplitude, were first determined from the signal acquired in free‐breathing mode. Then, a natural breathing sound that best matched the patient‐specific pattern was selected from a large database (900 sounds) and used for the audio coaching procedure. Furthermore, the patients saw their respiratory signal in real time, thanks to video‐glasses (eMagin Z800 3DVisor, Bellevue, WA). This visual feedback helped them to keep their breathing amplitude within a predefined range corresponding to the end‐exhalation and end‐inhalation of their averaged respiratory cycle.

Coaching was then used during all subsequent 4D CT acquisitions in order to evaluate its impact in terms of intersession reproducibility. The individual patient's capability of adequately understanding and following the real‐time visual feedback from his/her breathing was the criterion to select patients for AC alone (n=5) or combined A/VC (n=8). When the patient felt uncomfortable with visual feedback, which resulted in unstable breathing period and/or amplitude, only AC was applied. The respiratory signal was measured from the epigastric region using magnetic sensors (Nomics, Liège, Belgium).^(14)^ This system consists of a pair of magnetic probes, an emitter and a receptor, which measure their distance with an accuracy of 0.1 mm in a range from 3 to 10 cm. In our protocol, a maximum distance of 7 cm between the probes was allowed, such that magnetic interferences during 4D CTs remained negligible. A Plexiglas arch was designed in‐house and used to adequately position and hold the sensor receptors.

CT images were acquired on either a combined PET‐CT scanner (Gemini TF, Philips Medical System, Cleveland, OH), or a big bore CT scanner (Aquilon LB, Toshiba Medical System Corporation, Japan). First, a contrast‐enhanced CT (CE‐CT) was acquired in free breathing for the purpose of delineation (only for the first preparatory imaging session). Then, the 4D CT started using an axial FOV of 600 mm and a slice thickness of 2 mm. In 4D acquisition mode, the CT scanner automatically sets the optimal helical pitch according to the patient's breathing period measured by the magnetic sensors. The position of the magnetic probe on the skin was marked with a permanent felt‐tip pen in order to ensure reproducible positioning through the successive 4D CTs.

### C. Tumor motion and baseline shift analysis

The 4D CTs images were retrospectively phase‐sorted in ten equally distributed temporal bins, which were subsequently used to estimate the TM, as described here. First, an experienced physician delineated the gross target volume (GTV) on the CE‐CT. Then, nonrigid registration, relying on a diffeomorphic morphon algorithm,^(15)^ was used to propagate this contour to the ten respiratory CT phases derived from the 4D CT.^(16)^ Registration accuracy was visually checked for all phases. Finally, the center of mass of the GTV was calculated at each respiratory phase to determine the three‐dimensional TM (i.e., the tumor trajectory). The intersession reproducibility of the TM shape was then evaluated by Pearson correlation coefficient (CC) for each patient.

Tumor baseline shifts (BS) between the various 4D CTs were quantified using the MidP approach. The MidP CT was derived from each 4D CT according to Wolthaus et al.^(3)^ Basically, the average of the ten nonrigid deformation vector fields (DVF) calculated from the diffeomorphic morphon algorithm is subtracted from the ten primary DVFs. Each resulting DVF is then applied to its corresponding CT phase. Finally, the average of these CT phases leads to the MidP CT. Then, for each patient, all MidP CTs were rigidly aligned with the corresponding reference MidP CT (i.e., the one derived from the first 4D CT) at the level of the third thoracic vertebra. Finally, the random and the systematic baseline shifts in the three dimensions were evaluated. The center of mass calculated from all GTV positions was considered as the reference tumor position to determine the random shift, by averaging the distances between each GTV position and the reference position. On the other hand, the systematic shift component was determined in two steps. First, each possible GTV is successively chosen as a reference and the average distance to all other GTVs is computed. Next, these distances are averaged over all possible reference GTVs to get the systematic shift.

### D. Analysis of the respiratory signal

**Figure 1:**
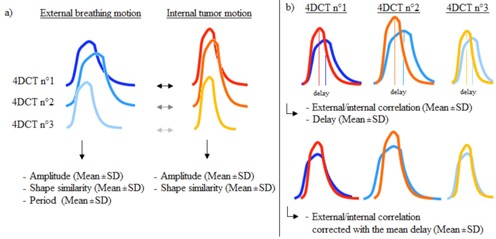
Illustration of the assessment of the internal/external motion reproducibility: (a) a single breathing cycle, for both external and internal motion, is computed in order to calculate the inter‐4D CTs variation of the amplitude, period, and shape similarity; (b) external/internal correlation and temporal delay between both signals have been measured for each 4D CT. Internal motion was subsequently corrected with the mean delay and external/internal correlation was computed again.

Only the segment of respiratory signal that was actually used for the reconstruction of CT slices including the tumor was analyzed. This prevented noisy and nonrepresentative respiratory cycles from being considered in the analysis. Variation in amplitude and period throughout the successive 4D CT acquisitions were analyzed, as well as the shape reproducibility assessed by Pearson correlation coefficients (see Fig.1(a)).

### E. Internal/external correlation

Correlation coefficients (CC) between TM in X, Y and Z directions and the respiratory signal were calculated. The phase difference between internal and external signals was determined with a cross‐correlation function (FC):^17^, ^18^

(1) $$FC_{d}=\sum_{n}^{N}T_{n}B_{n-d}$$

where $T_{n}$ and $B_{n}$ are the nth values of the sampled tumor and the breathing signals, respectively. The summation was performed over the total number of data points, N. Index *d* represents the delay between both signals. Maximizing correlation FC determines the delay where both signals are best aligned — that is, the phase shift. This phase shift was subsequently averaged over the repeated 4D CTs, in order to correct the signals and evaluate their “delay‐free” linear correlation.^(19)^ The corresponding internal/external CC was, therefore, computed for each 4D CT. For each patient, the mean CC and its variability were calculated (see Fig. 1(b)).

### F. Analysis of the residual tumor motion

The residual tumor motion (RTM) within the gating window was calculated with respect to the duty cycle (DC), defined as the ratio between the beam‐on time and the breathing period, for both types of methods (based on the displacement or phase).^(20)^ The standard deviation of the signal variation within the gating window was computed and used to describe the residual motion.^(11)^ The DC ratio varied from 10% to 100%, with steps of 10%. A value of DC equal to 100% corresponded to no gating at all.

**Figure 2:**
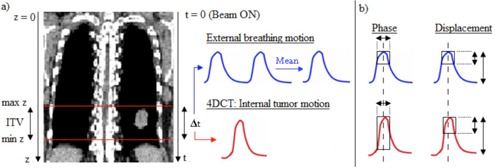
Illustration of the residual tumor motion (RTM) computation procedure: (a) the portion of the external breathing signal used for the 4D TM reconstruction is averaged to define the external signal upon which the gating window is performed; (b) the gating window is centered on the end of expiration (EE) and the corresponding residual TM is computed for both phase‐ and displacement‐based gating techniques. The phase‐based technique considers the RTM in the phase interval defined as DC% of the internal signal period while, for the displacement‐based method, the RTM is regarded as DC% of the amplitude of the internal signal. The smallest RTM was sought, since it leads to smaller margins to cover the motion within the gating window.

Intrasession variations of the external signal baseline are known to jeopardize phase‐based gating approaches.^(11)^ These variations could not be considered in this study since the 4D CT represents the TM over one breathing cycle. Consequently, the portion of the external signal used for the 4D TM reconstruction (typically one or two respiratory cycles) was averaged, too (see Fig. 2). The gating window was defined on the external signal, for each 4D CT session, centered on the end of expiration (EE) or on the end of inspiration (EI), and the corresponding 3D RTM (i.e., the residual motion within the gating window) was calculated for each DC. For each patient, 3D RTMs derived from each 4D CT were normalized by the 100% DC 3D RTM and averaged using their quadratic mean (Eq. (2)).

(2) $$3DRTM=\sqrt{\left(\sum_{i=1}^{N}{\left(3DRTM_{i}/3DTM_{i}\right)}^{2}\right)}$$

where *N* is the number of acquired 4D CTs for each patient, $3D\;{RTM}_{i}$ is the 3D standard deviation of the signal variation within the gating window from the ith 4D CT, and $3D\;{TM}_{i}$ is the 100% DC $3D\;{RTM}_{i}$ (i.e., the standard deviation of the signal variation from the ith 4D CT).

## III. RESULTS

The key characteristics of the observed breathing patterns are summarized in Tables 1 and 2. Results were analyzed separately for AC and A/VC to investigate the potential difference between combined audio‐visual guidance and audio coaching alone. Peak‐to‐peak amplitudes of the breathing motion, averaged for each patient over the three or four acquisitions, ranged from 7.1 to 19.9 mm, while TM amplitude ranged from 3.9 to 10.3 mm, 0.7 to 6.3 mm, and 0. 4 to 3.7 mm for SI, AP, and LR directions, respectively. As expected, A/VC led to improved reproducibility of the breathing amplitude through successive sessions than audio coaching alone (range and mean SD of 0.5‐2.2 and 1.2 vs. 1.6‐6.4 and 3.7, respectively). This favorably affected internal TM itself, which demonstrated reduced variations of amplitude 7.2 mm±1 vs. 8 mm±1.8, baseline shift (mean random errors: 1.2 vs. 2.2 mm, and mean systematic errors: 1.8 vs. 3.3 mm), and shape (mean CC of 0.96 vs. 0.91).

**Table 1:** Characteristics of breathing and tumor motion, and their intersession variations (±1 SD) for AC patients

		*P6*	*P8*	*P10*	*P12*	*P13*	*Mean*
Age (y)		70	75	81	60	85	74.2
Gender		M	M	M	M	M	/
Breathing Motion	Amplitude (mm)	14.49±1.56	10.81±3.62	18.89±2.29	10.51±2.36	11.62±6.41	13.3±3.7
	Period (sec)	3.90±0.14	3.28±0.06	4.33±0.27	3.53±0.26	4.00±0.14	0.96±0.02
	Shape (CC)	0.98±0.01	0.97±0.02	0.97±0.01	0.95±0.03	0.94±0.02	3.9±0.2
Tumor Motion (SI	Amplitude (mm)	10.16±0.50	6.24±2.39	10.15±0.87	6.03±2.03	10.31±2.46	8.6±1.8
	Shape (CC)	0.97±0.02	0.96±0.03	0.98±0.01	0.94±0.04	0.72±0.21	0.91±0.11
Baseline Shift (3D)	Random error (mm)	1.05±0.95	3.11±2.07	1.82±0.86	3.29±1.73	1.64±1.23	2.2±1.5
	Systematic error (mm)	1.55±1.45	4.56±3.09	2.72±1.29	4.93±2.60	2.46±1.84	3.3±1.3

Intersession variations of the internal/external CC and the mean delay between internal and external motions are shown in Table 3. For these endpoints, no relevant difference has been noticed between AC and A/VC patients for the SI direction, but less reproducible correlations have been observed when no visual feedback is performed.

**Figure 3:**
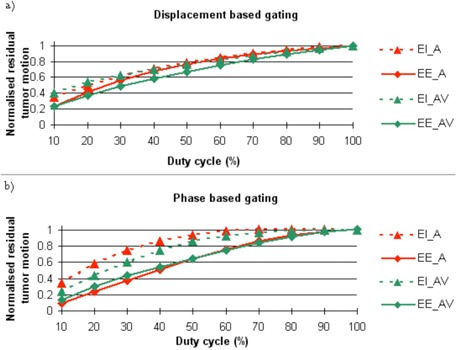
Standard deviation of the normalized residual tumor motion for: (a) displacement‐based gating and (b) phase‐based gating centered on the end of expiration (EE) and on the end of inspiration (EI), comparing patients who performed audio coaching (AC) or audio and visual coaching (A/VC), for duty cycle for 10% to 100%.

Finally, our results showed that smaller RTM were achieved at the end‐expiration, compared to the end‐inspiration, regardless which gating strategies or duty cycles were considered (Fig. 3). This confirms that gating strategies should preferably target the end of expiration since smaller RTM will translate into smaller PTV margin. By stabilizing breathing and TM amplitudes through sessions, A/VC slightly reduced the RTM for a displacement‐based gating approach.

**Table 2:** Characteristics of breathing and tumor motion, and their intersession variations (±1 SD) for A/VC patients

*P1*	*P2*	*P3*	*P4*	*P5*	*P 7*	*P9*	*P11*	*Mean*
Age (y)		50	74	77	79	46	50	70	75	65.1
Gender		M	M	M	M	M	F	M	M	/
Breathing Motion	Amplitude (mm)	15.48±2.24	17.00±1.00	19.93±1.05	17.27±0.76	20.12±1.13	7.09±0.48	10.85±0.64	11.63±1.25	14.9±1.2
	Period (sec)	5.05±0.07	6.32±0.21	4.88±0.04	4.83±0.21	4.46±0.06	3.63±0.05	4.55±0.48	3.03±0.21	0.96±0.02
	Shape (CC)	0.97±0.02	0.98±0.01	0.91±0.00	0.95±0.03	0.96±0.03	0.98±0.01	0.97±0.02	0.94±0.05	4.6±0.2
Tumor Motion (SI)	Amplitude (mm)	3.83±.62	8.54±2.09	6.44±0.46	8.32±1.11	10.27±0.88	6.27±0.98	5.50±0.18	8.53±1.06	7.2±1.0
	Shape (CC)	0.97±0.01	0.94±0.06	0.98±0.00	0.98±0.01	0.99±0.00	0.91±0.08	0.97±0.01	0.96±0.02	0.96±0.03
Baseline Shift (3D)	Random error (mm)	0.99±0.58	0.99±0.64	0.95±0.66	0.65±0.32	1.65±1.19	0.87±0.63	1.87±1.37	1.75±1.00	1.2±0.7
	1.49±0.87	1.49±0.96	1.36±0.97	0.97±0.48	2.50±1.50	1.36±0.68	2.80±2.05	2.63±1.50	1.8±1.0

**Table 3:** Mean value and intersession variations (±1 SD) of the mean delay, and the internal/external CC with and without delay correction

*Tumor Motion Direction* ^a^	*Mean Delay (sec)*		CC *(no delay correction)*		CC *(delay correction)*	
	*AC*	*A/VC*	*AC*	*A/VC*	*AC*	*A/VC*
SI	0.31±0.10	0.22±0.14	0.81±0.09	0.81±0.08	0.98±0.02	0.97±0.02
AP	0.49±0.21	0.45±0.08	0.54±0.16	0.50±0.14	0.84±0.08	0.90±0.05

a ^a^ CC are evaluated for TM direction where amplitude >5 mm. Only three patients (P11, P12, P13) fulfilled this condition for SI and AP directions, respectively.

## IV. DISCUSSION

Our study aimed to assess the difference between audio coaching alone and audio coaching complemented with real‐time visual feedback, in terms of lung TM reproducibility. In that regard, our study was the first to evaluate the potential gain of a visual feedback on the intersession reproducibility of the actual internal TM measured from repeated 4D CTs, and its correlation with the external surrogates of respiratory motion measured by magnetic sensors. Indeed, previous studies on respiratory coaching assessed either the impact of audio‐visual coaching on the breathing signal alone,^10^, ^11^ or looked at the internal motion itself but with audio guidance exclusively.^(9)^

The intersession reproducibility of the tumor trajectory and baseline position between treatment planning and delivery is an essential prerequisite for the clinical implementation of any RT strategy aimed at dealing with motion, especially when offline and/or bony‐based setup correction protocols are used. Indeed, the uncertainties of daily tumor positioning lead either to poor tumor coverage if they are neglected or to large safety margins if they are accounted for. In that regard, our study showed that combining visual feedback with audio coaching improved the stability of both the breathing and SI TM patterns through successive acquisitions, and resulted in similar reproducibility of the correlations between those two signals, compared to the Nakamura study^(9)^ in which the authors nonetheless performed only audio coaching. Moreover, investigation of correlation reproducibility for the AP direction has also been analyzed and seems better when coalescing audio and visual feedback.

Of interest is our method of audio guidance involved a breathing sound, which set the respiratory period and provided nonverbal, uninterrupted inspiration/expiration directives with smooth transitions. This proved to be more natural and comfortable for patients, in contrast with common verbal directives (“breathe in”, “breathe out”), which involve language skills and more complex cognitive processing. These features have certainly contributed to improve breathing and TM characteristics reproducibility. The variation of TM amplitude observed in our study (SD=1.8 mm in SI direction) for audio‐coached patients was lower compared to those previously reported by Nakamura et al.^(9)^ and Haasbeek et al.^(21)^ Similarly, we achieved CC values higher than 0.9 for the tumor trajectory shape for 12 out of 13 patients, while similar results were observed in only 10 out of 23 patients in the Michalski study.^(22)^

As demonstrated earlier, adding visual feedback to audio guidance improves the stability of the TM amplitude, with small residual variations (SD=1 mm for SI direction). Interestingly, A/VC might possibly improve the reproducibility of the internal‐external correlation through sessions, since it has been reported to depend on motion amplitude variability itself.^9^, ^19^ In addition, A/VC apparently reduced the variations of phase delay in the AP direction, when compared to data without A/VC that reached higher maximum variations (0.4‐0.6 s vs. 0.18 s).^(19)^

In the framework of a gated treatment strategy, our data confirm that the gating window should ideally be centered on the end of expiration, which is the most stable phase of breathing, as previously shown by George et al.^(11)^ We furthermore recommend the use of A/VC if a displacement‐based gating approach is considered. Indeed, thanks to its ability to stabilize the breathing and TM amplitudes through successive sessions, A/VC led to smaller residual TM within clinically relevant duty cycles ranging from 10% to 40%, compared to AC alone. We should, however, consider this statement with caution, the impact of possible intrasession shifts of the tumor baseline having not been integrated in our model (data not available). However, we can reasonably anticipate that small baseline shifts would occur during a given session with our configuration, since these shifts were already limited between different sessions (random and systematic shifts of 2.6 and 5 mm), and were smaller than previously reported.^23^, ^24^, ^25^

The main drawback of this study is the lack of a direct comparison between various coaching strategies within the same population of patients, which would have allowed us to draw unbiased conclusions about the added value of A/VC with respect to AC alone and free‐breathing approaches. As the main endpoint of this study was the assessment of the tumor motion through successive sessions, the ideal study design would have required an unacceptable high number of 4D CTs per patient (three to four 4D CTs per strategy). This imaging modality being highly irradiating, it inherently restricts the sampling possibilities to a few imaging sessions per patient. We thus stratified our patients between A/VC and AC on a pragmatic basis (i.e., their individual capability of adequately following real‐time visual feedback). We have indeed observed that some patients, as well as some healthy volunteers, struggled to synchronize their breathing with simultaneous audio and visual directives, while they felt more comfortable with audio coaching alone. This probably reflected the inability of some subjects to understand complex instructions and to reproduce them accurately, which occurred more often in older patients (mean age of 74.2 y and 65.1 y for AC and A/VC patient groups, respectively). This also means that A/VC is not applicable to all patients, even if a longer training session might potentially extend the use of A/VC to a larger population. Fortunately, patients from both groups shared similar breathing and tumor motions (Tables 1 and 2), and therefore we believe that our observations remain valid. However, they should be further confirmed on a larger population before drawing any definitive conclusion. Another limitation was that some tumor motions were still blemished, despite the use of A/VC, by inherent artifacts associated with the finite time resolution of 4D CT reconstruction technique.

## V. CONCLUSIONS

Our data suggest that audio and visual coaching might improve the reproducibility of key characteristics of both breathing and tumor motion patterns through multiple fractions, including the amplitude, period, and shape. Coaching might thus be considered for the clinical implementation of lung tumor radiotherapy.

## ACKNOWLEDGMENTS

This research program was supported by two grants from Wallonia (convention numbers 6066 ‐ RPI and 1017266 ‐ InvivoIGT) and the MecaTech Cluster. Xavier Geets is a postdoctoral researcher partly funded by the FNRS (grant number, 3.4600.08). John A. Lee is a Research Associate funded by the FNRS. Guillaume Janssens is a postdoctoral researcher funded by Wallonia.
